# Unsuspected Malignancy During Percutaneous Nephrolithotomy: The Snake in the Grass

**DOI:** 10.1089/cren.2016.0110

**Published:** 2016-11-01

**Authors:** Charlotte Q. Wu, Justin T. Matulay, Mantu Gupta, Piruz Motamedinia

**Affiliations:** ^1^ Department of Urology, Yale University, New Haven, Connecticut.; ^2^ Department of Urology, New York-Presbyterian Columbia University, New York, New York.; ^3^ Department of Urology, Ichan School of Medicine at Mt. Sinai, New York, New York.

**Keywords:** squamous cell carcinoma, nephrolithiasis, UT-SCC

## Abstract

Squamous cell carcinoma of the upper tract (SCC-UT) is a rare neoplasm that disproportionately affects patients with longstanding nephrolithiasis. Diagnosis is challenging and typically comes at late stages; as such, the prognosis is poor. The absence of a reliable diagnostic predictor for SCC highlights the need to keep the diagnosis in mind for at-risk patient populations. In this study, we describe a small case series of rapidly progressive SCC-UT incidentally discovered during percutaneous nephrolithotomy.


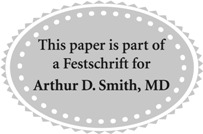


## Introduction

Squamous cell carcinoma (SCC) of the upper urinary tract (SCC-UT) is a rare aggressive malignancy, with a reported prevalence of <1% of all urinary tract neoplasms.^1^ SCC-UT is associated with chronic inflammation of the renal pelvis such as recurrent infection and in longstanding stone disease. Diagnosis typically comes at late stages, and prognosis is poor. As the prevalence of stone disease is increasing worldwide, it is important to keep this malignancy in the differential when evaluating at-risk patients.^2^ In this study, we present two cases of rapidly progressive SCC-UT incidentally discovered during percutaneous nephrolithotomy (PCNL).

## Case 1

A 77-year-old male presented to the urology clinic in August 2011 with voiding symptoms and a known history of nephrolithiasis. A CT of the abdomen and pelvis demonstrated bilateral extensive nephrolithiasis with a large left staghorn renal calculus, 3 cm bladder stone, and a markedly enlarged prostate. The patient was recommended surgery for the bladder and renal stones although was lost to follow-up.

**FIG. 1.**
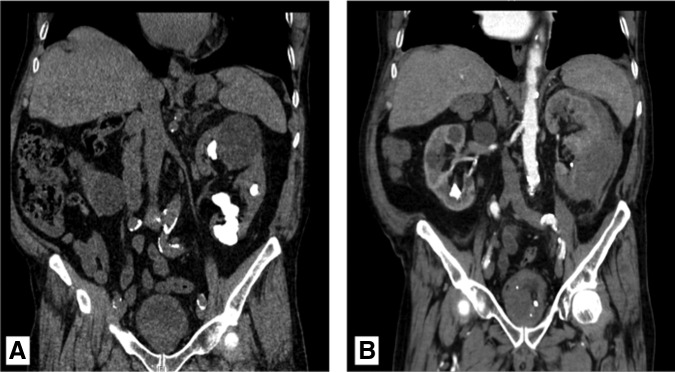
Case 1: Interval development of a left renal mass. **(A)** CT abdomen pelvis from July 2013 demonstrating stable stone burden compared to the original CT from August 2011 (not shown). **(B)** CT scan in September 2013 following PCNL, revealing a subcapsular hematoma and heterogeneous density in the left renal pelvis concerning for tumor *vs* blood clot. PCNL, percutaneous nephrolithotomy.

He re-presented 22 months later and repeat noncontrast CT abdomen and pelvis revealed stable appearance of his large left staghorn stone, right kidney stones, and bladder stone (Fig. 1A). New diffuse bladder wall thickening was also noted. Transurethral resection of the prostate and cystolitholapaxy were performed along with bladder biopsy given CT findings. Prostate pathology demonstrated focal squamous metaplasia and the bladder showed atypical squamous proliferation with areas of necrosis—both consistent with chronic inflammation.

He subsequently underwent left PCNL with removal of the staghorn calculus. However, an incidental renal pelvis mass was encountered. The mass was biopsied and resected using a bipolar resectoscope. The immediate postoperative course was complicated by acute blood loss anemia requiring blood transfusions and angioembolization of the left kidney. Pathology ultimately revealed multifocal, invasive poorly differentiated SCC of the left renal pelvis with spindle cell features. Renal parenchyma was seen without carcinoma invasion.

A restaging CT abdomen and pelvis demonstrated a pseudoaneurysm of the left kidney, residual renal pelvis tumor, as well as significant bladder wall thickness concerning for tumor (Fig. 1). There was no evidence of lymph node involvement or metastatic disease.

Cystoscopic evaluation of the bladder revealed diffuse tumor. Tumor resection pathology revealed high grade SCC invading the lamina propria without muscle invasion.

An attempted robotic left nephroureterectomy with cystoprostatectomy and urinary diversion was performed ∼3 months following PCNL. The delay in definitive management was attributed to the need for further staging and medical optimization for major surgery. The intraoperative course was complicated by a difficult dissection and hemodynamic instability following nephroureterectomy. Unfortunately, the procedure was aborted before cystectomy and lymph node dissection.

The left kidney and ureter pathology revealed pT4NX disease for invasion into the ipsilateral adrenal gland. The invasive SCC demonstrated sarcomatoid features. Also noted was focal intestinal metaplasia of the renal pelvic epithelium.

**FIG. 2.**
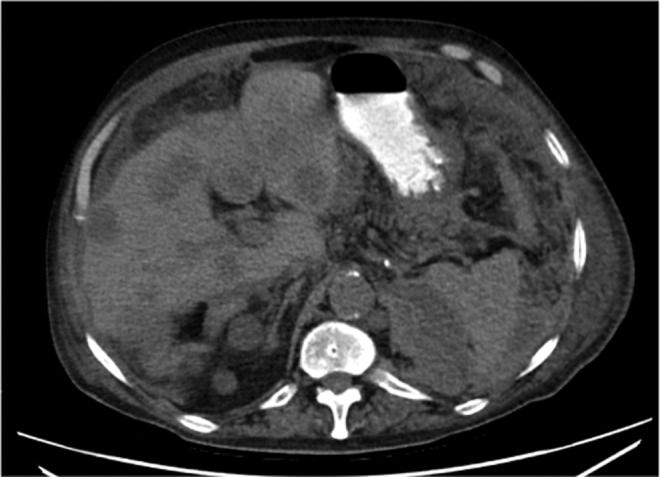
Case 1: CT scan from January 2014 following left nephroureterectomy demonstrating diffuse new hypoattenuating lesions consistent with metastatic disease. There is also peritoneal carcinomatosis, increased lymphadenopathy, and moderate intra-abdominal ascites.

His postoperative course was complicated by acute blood loss anemia and severe sepsis. A CT of the abdomen and pelvis (3 months following last staging CT) demonstrated increased lymphadenopathy, and new lesions throughout the liver with peritoneal carcinomatosis consistent with metastatic disease (Fig. 2). The patient expired 21 days following surgery.

## Case 2

**FIG. 3.**
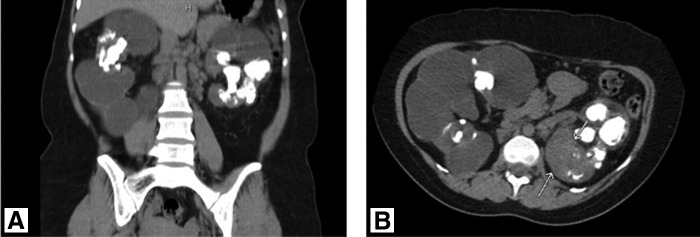
Case 2: Original preoperative CT scan from June 2015 (**A**: coronal, **B**: axial) demonstrating bilateral staghorn calculi and a hyperdensity in the central region of the left kidney (*arrows*) felt to represent indeterminate, possibly representing hemorrhagic or proteinaceous debris.

A 54-year-old woman with renal insufficiency (GFR 16 mL/minute/1.73 m^2^) and known kidney stones, who had deferred definitive stone management for nearly 5 years, presented to our clinic in June of 2015 with left flank pain. A CT scan of the abdomen demonstrated bilateral staghorn calculi, severe bilateral hydronephrosis, and minimal right renal parenchyma with thinned left sided parenchyma. Compared to prior CT in 2013, there was an additional finding of a new hyperdensity within the left lower pole suggestive of hemorrhagic or proteinaceous debris (Fig. 3). She complained of increased fatigue and pain, however, denied weight loss or hematuria. She was desirous of definitive left stone removal for renal preservation and pain control and was counseled toward a staged PCNL.

After gaining percutaneous access and removing a moderate volume of stone, we noted abnormal shaggy appearing urothelium of the collecting system, which was biopsied. The intraoperative course was complicated by moderate blood loss hypotension. The patient required postoperative blood transfusions and inotropic support and, ultimately, required interventional radiology guided angiogram and selective renal artery embolization to cease blood loss. Renal function declined as a result of the embolization, intravenous contrast nephropathy, and acute blood loss anemia and the patient ultimately required hemodialysis. After stabilization, she was discharged on postoperative day 13.

**FIG. 4.**
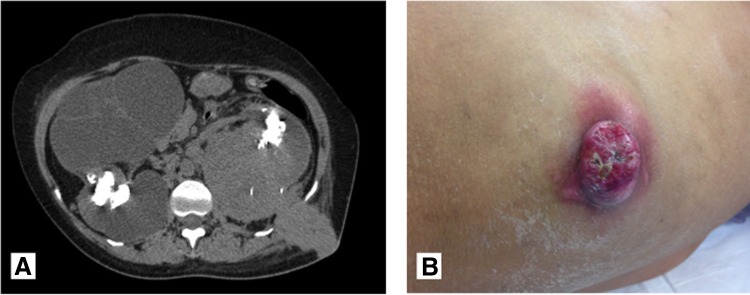
Case 2: Flank mass eruption from the skin at the nephrostomy tube site. **(A)** CT abdomen pelvis performed 1 month following PCNL demonstrating what was felt to be hematoma extending to the skin. Nonspecific para-aortic lymph node enlargement was also noted. **(B)** One week following CT scan, the patient developed eruption of a mass-like structure from the nephrostomy tube site. Biopsy revealed well-differentiated keratinizing SCC.

Histologic assessment of the inflammatory tissue revealed papillary proliferation with squamous metaplasia and moderate atypia, interpreted as reactive urothelium given long-standing stone history. At 1-month follow-up, physical examination revealed a tender cutaneous bulge on her left flank, at the site of her previous nephrostomy, concerned for hematoma or abscess. A CT of the abdomen favored hematoma extending to the skin (Fig. 4A) and also noted nonspecific para-aortic lymph node enlargement. Shortly after, the collection was noted to erupt through her flank, revealing a likely tumor (Fig. 4B). A biopsy of the mass revealed a well-differentiated keratinizing SCC. The patient underwent biopsy of the para-aortic lymph nodes, which were positive for metastatic carcinoma of urothelial primary, confirming stage IV disease. Further radiographic staging was negative for visceral metastasis; however, there was interval growth of the renal mass, now replacing the majority of the left kidney, extending through her flank and inferiorly to the psoas.

**FIG. 5.**
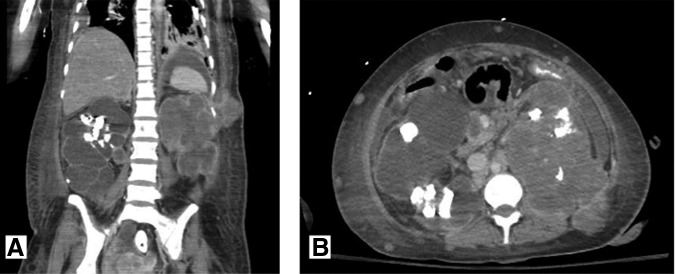
Case 2: CT abdomen and pelvis from December 2015 (**A:** coronal, **B:** axial) demonstrating marked progression of metastatic disease, including metastatic lesions of the lung, pleura, pancreatic neck, and innumerable lesions in the subcutaneous tissue.

Further management of this patient was discussed at a multidisciplinary tumor board, which recommended radiation and chemotherapy; however, she refused further treatments in favor of palliative care. She re-presented to the hospital 1 month later with hypotension and fever suggesting urosepsis. A CT of the chest, abdomen, and pelvis with contrast demonstrated metastatic lesions of the lungs, pleura, pancreatic neck, and innumerable metastatic lesions in the subcutaneous tissues (Fig. 5). Flank mass was now over 13 cm. She expired 3 days later.

## Discussion

Squamous cell carcinoma of the kidney or renal pelvis is rare comprising only 0.3%–0.8% of kidney tumors.^3,4^ Much of what is known about SCC of the renal pelvis comes from isolated case reports and small case series. While rare among the general population, the diagnosis is nearly exclusive to a subset of patients with a history of renal stones, obstruction/hydronephrosis, or infection, with nephrolithiasis present in as much as 50% of patients.^5^

Histogenesis of pure SCC of the urinary tract occurs in two forms: malignant transformation of squamous metaplasia or extensive squamous differentiation of transitional-cell carcinoma.^6,7^ In the renal pelvis, pure SCC is often seen with squamous metaplasia of the nearby urothelium.^8^ On histology, squamous differentiation is characterized by intercellular bridges and keratinization. SCC may be evident by atypical squamous cells, highly atypical nuclei with irregular nuclear contour, abundant mitotic figures, and invasion into the underlying renal parenchyma.

The patients presented in this case series exhibited many similarities, including large renal stone with presumably a chronic inflammation, bilateral hydronephrosis, and ultimately, a moderate delay to definitive treatment. Despite preoperative cross-sectional imaging in both cases, the tumor was discovered incidentally during PCNL, and with some index of suspicion of intraoperative findings. Final pathology was needed to confirm diagnosis and following diagnosis there was a rapid progression to death (4 and 2 months in Case 1 and 2, respectively).

Early diagnosis and detection of SCC continues to be challenging, although appears to be imperative if a poor outcome is to be averted. A number of studies have demonstrated a 5-year survival for SCC of less than 10%, due largely to late stage diagnosis; over 95% of SCCs are diagnosed at stage pT3 or higher.^1^ Surveillance, Epidemiology and End Results (SEER) Program data from the National Institutes of Health describe a 5-year survival rate of 4% for SCC, the lowest among all renal and renal pelvis malignancies, compared to 57.5% of urothelial carcinoma.^4^ This is in large part to the advanced stage at diagnosis. When SCC and urothelial carcinoma of the upper tract were compared stage for stage, the prognosis was not different.^1^

Immunostaining profile may be of use for a tumor that is otherwise challenging to diagnose. Pure SCCs are typically positive on immunostains for CK14 and desmoglein-3 and typically negative for GATA-3, uroplakin-III, and S100-P.^9^ These latter three are typical of “urothelial” staining pattern and in urothelial carcinoma with squamous differentiation, their expression is reduced compared to pure urothelial carcinomas.^9,10^ Also described in the literature is a potential biomarker for urine screening of patients with SCC. In limited studies, the calcium binding protein, psoriasin, typically produced by stratified squamous epithelia, was identified in the urine of four patients with bladder SCC and later identified in six additional patients harboring bladder SCC.^6,11^ Whether psoriasin is also expressed in the urine of patients with renal pelvis SCC is worthy of investigation.

In patients who do present with late stage SCC, treatment options are limited. The mainstay of treatment is nephrectomy or nephroureterectomy. Given the unifocality of the disease, it has been suggested that nephron or parenchyma sparing surgery such as partial nephrectomy or partial ureterectomy can be performed.^5^ Platinum-based adjuvant chemotherapy can also be implemented, as well as radiation, although the disease typically progresses despite these.

The question remains: what if anything can be done to improve early detection of SCC? Imaging characteristics such as a renal mass, hydronephrosis, and calcifications are nonspecific.^3,12^ Symptoms such as pain and hematuria, when present are also nonspecific and are commonly attributed to the underlying stone disease or infection, which is also common to these patients. Contrast enhanced imaging to better characterize potential soft-tissue masses may be helpful, but is often contraindicated in patients with renal insufficiency, as in case 2. For patients who present with a flank mass or abnormal abdominal examination, or other symptoms, including anorexia, cachexia, or hematuria, the disease is likely in a more advanced stage. Unfortunately, the first indication for malignancy is commonly an incidental finding, described in several prior case reports.^13^ The absence of a reliable diagnostic predictor for SCC highlights the need to keep the diagnosis in mind for at-risk patient groups. Any suspicious finding potentially representing tumor should be thoroughly investigated with biopsy, histopathologic assessment, and immunohistochemical stain profiling. Heroic efforts at stone removal in the setting of aggressive malignancy should be tempered against the risk of hemorrhagic and metastatic complications that patients likely need for radical surgery and poor oncologic prognosis.

## Abbreviations Used

CT: computed tomography
PCNL: percutaneous nephrolithotomy
SCC: squamous cell carcinoma
SCC-UT: squamous cell carcinoma of the upper tract
